# Food–Drug Interaction between the Adlay Bran Oil and Drugs in Rats

**DOI:** 10.3390/nu11102473

**Published:** 2019-10-15

**Authors:** Hsien-Tsung Yao, Jia-Hsuan Lin, Yun-Ta Liu, Mei-Ling Li, Wenchang Chiang

**Affiliations:** 1 Department of Nutrition, China Medical University, 91 Hsueh-shih Road, Taichung 404, Taiwan; chlin@leesclinic.org (J.-H.L.); hhh12324@msn.com (Y.-T.L.); u104059001@cmu.edu.tw (M.-L.L.); 2 Graduate Institute of Food Science and Technology, Center for Food and Biomolecules, College of Bioresources and Agriculture, National Taiwan University, 1 Roosevelt Road, Sec. 4, Taipei 106, Taiwan; chiang@ntu.edu.tw

**Keywords:** adlay, adlay bran oil, cytochrome P450, food–drug interactions, rats

## Abstract

Adlay (*Coix lachryma-jobi* L. var. *ma-yuen* Stapf) contains various phytonutrients for treating many diseases in Asia. To investigate whether orally administered adlay bran oil (ABO) can cause drug interactions, the effects of ABO on the pharmacokinetics of five cytochrome P450 (CYP) probe drugs were evaluated. Rats were given a single oral dose (2.5 mL/kg BW) of ABO 1 h before administration of a drug cocktail either orally or intravenously, and blood was collected at various time points. A single oral dose of ABO administration did not affect the pharmacokinetics of five probe drugs when given as a drug cocktail intravenously. However, ABO increased plasma theophylline (+28.4%), dextromethorphan (+48.7%), and diltiazem (+46.7%) when co-administered an oral drug cocktail. After 7 days of feeding with an ABO-containing diet, plasma concentrations of theophylline (+45.4%) and chlorzoxazone (+53.6%) were increased after the oral administration of the drug cocktail. The major CYP enzyme activities in the liver and intestinal tract were not affected by ABO treatment. Results from this study indicate that a single oral dose or short-term administration of ABO may increase plasma drug concentrations when ABO is given concomitantly with drugs. ABO is likely to enhance intestinal drug absorption. Therefore, caution is needed to avoid food–drug interactions between ABO and co-administered drugs.

## 1. Introduction

Cytochrome P450 (CYP) enzymes are major phase I monooxygenases that catalyze the oxidative metabolism of various drugs, toxic chemicals, and many endogenous substrates. About 90% of human drug oxidation is attributed to six main CYP enzymes: CYP1A2, 2C9, 2C19, 2D6, 2E1, and 3A4 [1]. The method of administering a specific probe drug and then measuring the plasma kinetics of the probe has been widely used to estimate CYP isozyme activity in vivo [2]. CYP metabolic activity can be assessed before or during pharmacotherapy to adjust individual drug doses. For convenience, to reduce costs, and to accelerate our understanding of various CYP activities in vivo, a “cocktail” approach has been used as a screening tool for potential food/herb–drug interactions [3,4,5,6]. Recently, food/herb–drug interactions have become an important issue in health care. Induction or inhibition of CYP activities by phytochemicals, especially flavonoids and phenolic acid, present in functional foods and herbal medicines can change the pharmacological activities and toxicities of drugs [7,8]. Grapefruit juice–drug interactions are a well-known example of a food–drug interaction that can significantly increase the oral bioavailability of various medications. The likely mechanism of this increase is the inhibition of CYP enzymes, especially CYP3A4 in the liver and small intestine [9,10,11,12]. Naringin, an abundant flavonoid in grapefruit juice, is regarded as a potent CYP inhibitor and can inhibit the metabolism of some drugs, mainly those catalyzed by CYP3A4 (e.g., midazolam, triazolam, terfenadine, cyclosporin) [1].

Adlay seed is a popular traditional medicinal food or traditional Chinese medicine in Asia. The adlay seed consists of four parts from outside to inside: the hull, testa, bran, and endosperm. Many parts of the adlay seed have been demonstrated to lower inflammation, hyperlipidemia, immune disorders, diabetes, hypertension, and hyperuricemic and neoplastic diseases [13,14,15,16,17,18]. The bran part of adlay contains abundant neutral oil (approximately 25% of the dry weight), which is mainly present in the form of triglyceride (>90%) [19,20]. Adlay bran ethanolic extract or adlay bran oil (ABO) contains various phytonutrients, including phytosterols, flavonoids (e.g., nobiletin, tangeritin, rutin, and quercetin) and phenolic acids [21]. Studies have shown that ABO may have pharmacological effects that aid in the prevention and treatment of many diseases [13]. For example, ABO supplementation in the diet (10%) for four weeks can reduce hyperlipidemia in normal and type 2 diabetic rats [18,20]. In addition, ABO can lower inflammation by lowering lipopolysaccharide-stimulated interleukin 6 (IL-6) and tumor necrosis factor-α (TNF-α) secretions in RAW264.7 cells and murine peritoneal macrophages [22]. A human study demonstrated that ABO administered orally can lower the risk for severe acute radiation dermatitis in patients with breast cancer undergoing radiotherapy [23].

In our previous study, we showed that ABO inhibited various CYP-catalyzed enzyme reactions in both rat and human liver microsomes in vitro and reduced CYP1A1, 1A2, 2C, 2D, 2E1, and 3A activities and protein expressions in rat liver after four weeks of feeding with ABO [21]. However, whether ABO can affect the plasma drug concentration when administered concomitantly with a drug remains unknown. In this study, rats were given a single oral dose of ABO or consecutive feeding with an ABO-containing diet for 7 days, and then were administered a drug cocktail to investigate the possible food–drug interaction between the adlay bran oil and five cytochrome P450 probe drugs, theophylline (CYP1A2), diclofenac (CYP2C), dextromethorphan (CYP2C), chlorzoxazone (CYP2E1), and diltiazem (CYP3A), in rats [24,25]. Results from this study showed that a single oral dose or short-term administration of ABO may increase plasma drug concentrations when ABO is given concomitantly with drugs. 

## 2. Materials and Methods 

### 2.1. Chemicals and Reagents 

Theophylline, dextromethorphan hydrobromide, diclofenac sodium salt, chlorzoxazone, (+)-cis-diltiazem hydrochloride, NADPH, and heparin were obtained from Sigma-Aldrich (St. Louis, MO, USA). Methoxyresorufin, p-nitrophenol, resorufin, testosterone, and 4-nitrocatechol were obtained from Sigma-Aldrich (St. Louis, MO, USA). Dextrophan, 6-hydroxychlorzoxazone, 4-hydroxydiclofenac, and 6-β-hydroxytestosterone were purchased from Ultrafine Chemicals (Manchester, UK). All other reagents and chemicals were of analytical grade and were obtained commercially. 

### 2.2. Preparation of Adlay Bran Oil 

Adlay seeds were purchased from local farmers who planted Taichung Shuenyu no. 4 (TCS4) of *Coix lachryma-jobi* L. var. *ma-yuen* Stapf in Taichung, Taiwan. Adlay bran was separated from dehulled adlay, blended into a powder, and screened through a 20-mesh sieve. Adlay bran powder was extracted with ethanol (1:6; w/v) and concentrated under reduced pressure by use of a rotary vacuum evaporator to obtain ABO, which was provided by Dr. Wenchang Chiang, National Taiwan University. In general, each gram of ABO was derived from 10 g of adlay bran powder. The phytonutrients in ABO included β-sitosterol (1700 μg/g), stigmasterol (443 μg/g), campesterol (970 μg/g), total phenols (3583 μg/g), 4-hydroxybenzoic acid (167.3 μg/g), nobiletin (54.6 μg/g), tangeritin (44.5 μg/g), rutin (41.2 μg/g), quercetin (26.4 μg/g), and other phenolic acids such as ferulic acid (27.5 μg/g) and vanillic acid (22.2 μg/g) [21]. ABO contained a considerable amount of neutral oil that was present in the form of triglyceride (>90%; w/w) as described above. Compositions of the fatty acids in ABO were 18.0% palmitic acid (C_16:0_), 48.1% oleic acid (C_18:1_), and 32.4% linoleic acid (C_18:2_) [21]. ABO was stored at −20 °C until further use.

### 2.3. Animals and Treatment 

Experiment I: To investigate whether a single oral dose of ABO could affect the plasma drug levels when administered of the drug cocktail intravenously (through the rat tail vein) or orally (via an intragastric tube). The drug cocktail consisted of five in vivo specific CYP probe drugs, including theophylline, diclofenac, dextromethorphan, chlorzoxazone, and diltiazem for the evaluation of CYP1A2, 2C, 2D, 2E1, and 3A isozyme activity, respectively [24,25]. Male Sprague–Dawley (SD) rats, weighing about 300 g each (8–10 weeks old) and cannulated (PE-50) in the jugular vein, were obtained from BioLASCO, Ilan, Taiwan. The five drugs were freshly prepared and then administered to 2 groups of 6 rats via intravenously (IV) or oral administration (PO) at a dose volume of 5 mL/kg body weight (BW), which contained theophylline (1 mg/kg BW for IV administration; 10 mg/kg BW for PO administration), diclofenac (10 mg/kg BW for IV administration; 20 mg/kg BW for PO administration), dextromethorphan (5 mg/kg BW for IV administration; 25 mg/kg BW for PO administration), chlorzoxazone (1 mg/kg BW for IV administration; 5 mg/kg BW for PO administration), and diltiazem (5 mg/kg BW for IV administration; 40 mg/kg BW for PO administration), in formulation of 5% DMSO/8% cremophore/87% H_2_O. In the pilot study, a single drug or the drug cocktail was administered intravenously or orally, and blood was collected at various time points for all five probe drugs. The results showed little influence on area under the plasma drug concentration curve (AUC) values for most probe drugs between the single drug and the drug cocktail administration. These results suggested that the administration of the drug cocktail may cause less metabolic interactions.

Some food–drug interactions involving CYP inhibition in the gastrointestinal tract were observed when the drug was taken with food or juice within 2 h [26]. In this study, rats were orally administered 2.5 mL/kg BW soybean oil (control oil, equivalent to 24.3 g/60 kg adult) or ABO 1 h before given the drug cocktail. The volume of dosing solution administered was adjusted according to the body weight recorded before dose administration. At 0 (prior to dosing), 2, 5, 15, and 30 min and at 1, 2, 4, 6, 8, and 12 h after dosing, blood samples (∼200 μL) were collected from each animal via the jugular-vein cannula (no blood collection for 2 and 5 min for PO administration of drug cocktail). The same volume of normal saline was administered to the rats via the jugular vein to compensate for the blood loss. After the collection of all of the blood samples, the animals were sacrificed by exsanguination via the abdominal aorta while under carbon dioxide (70:30; CO_2_/O_2_) anesthesia. Heparin was used as an anticoagulant. Plasma was separated from the blood by centrifugation (3000× *g* for 20 min at 4 °C) and the concentrations of the five drugs in plasma were simultaneously determined by high-performance liquid chromatography/mass spectrometer (HPLC/MS). The animals were maintained in accordance with the guidelines for the care and use of laboratory animals [27]. This study was approved (No: 102-70-N) by the Institutional Animal Care and Use Committee (IACUC) of China Medical University, Taiwan. 

### 2.4. Sample Preparation

Plasma (50 μL) was mixed with 100 μL of acetonitrile. The mixture was vortexed for 30 s and then centrifuged at 21,000× *g* for 20 min. An aliquot (40 μL) of the supernatant was used for HPLC/MS. To prepare the samples of the calibration curve, the blank plasma (50 μL) containing various concentrations of theophylline (100–15,000 ng/mL), diclofenac (50–15,000 ng/mL), dextromethorphan (2.67–2000 ng/mL), chlorzoxazone (2.5–1500 ng/mL), or diltiazem (1.67–1000 ng/mL) was mixed with 100 μL of acetonitrile.

### 2.5. HPLC/MS Analysis

The HPLC/MS system consisted of an Agilent 1100 Series LC System and a single quadrupole mass spectrometer (Palo Alto, CA, USA). The column used to analyze the probe drugs was a Zorbax Eclipse XDB-C8 (5 μm, 150 × 3.0 mm i.d., Agilent Technologies, Palo Alto, CA, USA). The mobile phase consisted of Solvent A (acetonitrile + 0.5% formic acid) and Solvent B (10 mM ammonia acetate + 0.5% formic acid). The flow rate was 0.5 mL/min. The gradient system used to separate the five drugs was as follows: 90% B (0–1 min), 90% B to 10% B (1–12 min), 10% B (12–15 min), and 10% B to 90% B (15–15.5 min). The total running time was 22 min. The injection volume was 40 μL. The positive selected ion monitoring (SIM) mode was used before 10.5 min; after that, the negative SIM mode was used. The retention times of the five drugs were as follows: theophylline, 5.0 min; dextromethorphan, 9.6 min; diltiazem, 9.8 min; chlorzoxazone, 11.2 min; diclofenac, 14.3 min. Ions representing the positive mode ([M-H]^+^: theophylline at *m/z* 181; dextromethorphan at *m/z* 272.4; diltiazem at *m/z* 415.5) or negative mode ([M-H]^-^: diclofenac at *m/z* 295; chlorzoxazone at *m/z* 168) were selected and the peak areas were measured. Plasma samples that had concentrations above the upper limit of quantitation were diluted proportionally with control plasma before extraction with acetonitrile. The concentrations of the five drugs in rat plasma were determined with the calibration curves of authentic standard.

Experiment II: To investigate whether short-term feeding with ABO affected the pharmacokinetics of the five CYP probe drugs and CYP activities in the liver and small intestine, male SD rats were fed a control diet or an ABO-containing diet for 7 days. Animals were fed an experimental diet containing 20% casein, 20% dietary oil (10% soybean oil + 10% olive oil for control group or 20% ABO for ABO group), 1% vitamin mixture, 4% mineral mixture, 0.2% choline chloride, 5% cellulose, and 49.8% corn starch. Compositions of fatty acids in lipid extracted from the control diet were 11.8% palmitic acid (C_16:0_), 46.8% oleic acid (C_18:1_), and 35.6% linoleic acid (C_18:2_). The corresponding values in the ABO diet were 17.9% palmitic acid (C_16:0_), 48.1% oleic acid (C_18:1_), and 32.4% linoleic acid (C_18:2_). The vitamin and mineral mixtures (AIN 93) were purchased from ICN Biochemicals (Costa Mesa, CA, USA). The rats were housed in individual cages in a room kept at a temperature of 23 ± 1 °C and relative humidity of 60% ± 5% with a 12 h light and dark cycle. After 7 days of ABO feeding, rats were fasted overnight and the same oral dose of the drug cocktail was administered to 2 groups of 6 rats. Blood samples (∼200 μL) were collected from each animal via the rat tail vein at 15 and 30 min and 1, 2, 4, 8, and 12 h. Then, rats were sacrificed and the plasma was collected as described above. Plasma drug concentration was determined by HPLC/MS as described above. The duodenum portion of the intestine was collected and incubated with ice-cold PBS buffer containing protease inhibitors (1 mM phenylmethylsulfonyl fluoride, 1 μg/mL leupeptin, 10 μg/mL pepstatin A, and 2.5 μg/mL aprotinin) for at least 5 min and then scraped with a glass slide over ice to remove the mucosa. The liver and intestinal mucosa were stored at −80 °C until further analysis.

### 2.6. Microsomes Preparation

The liver sample (1 g) was homogenized with 4 mL of ice-cold 0.1 M phosphate buffer (pH 7.4) containing 1 mM EDTA. The homogenates were centrifuged at 10,000× *g* for 15 min at 4 °C. The supernatant was then re-centrifuged at 105,000× *g* for 1 h at 4 °C. The resulting microsomal pellet was suspended in 0.25 M sucrose solution containing 1 mM EDTA and was stored at –80 °C until use. The mucosa removed from the duodenal portion was homogenized and the resulting homogenates were used to prepare microsomes using the same method as described above.

### 2.7. CYP Enzyme Activity Assays

Activities of several CYP enzymes in microsomes isolated from the liver or small intestine were determined as reported previously [21]. Methoxyresorufin (5 μM) was used as the probe substrate for methoxyresorufin O-demethylation (CYP1A2), and diclofenac (4 μM), dextromethorphan (5 μM), p-nitrophenol (50 μM), and testosterone (60 μM) were respectively used as the probe substrates for diclofenac 4-hydroxylation (CYP2C), dextromethorphen O-demethylase (CYP2D), p-nitrophenol 6-hydroxylation (CYP2E1), and testosterone 6β-hydroxylation (CYP3A). Microsomal proteins (0.2 mg/mL) and the incubation time (15 min) were the same for all metabolic reactions. The metabolites of each CYP enzyme reaction were determined by HPLC/MS methods as reported previously [28]. 

### 2.8. Statistical Analysis 

For the pharmacokinetic study, WinNonLin software program (version 3.1, Pharsight, CA, USA) was used to analyze plasma concentration data using the standard non-compartmental method. Statistical differences between groups in two animal studies were analyzed by using one-way ANOVA (SAS Institute, Cary, NC, USA). The differences were considered to be significant at *p* < 0.05 as determined by independent-sample *t*-tests.

## 3. Results

### 3.1. HPLC/MS Chromatograms of the Five CYP Probe Drugs in Rat Plasma

**Figure 1:**
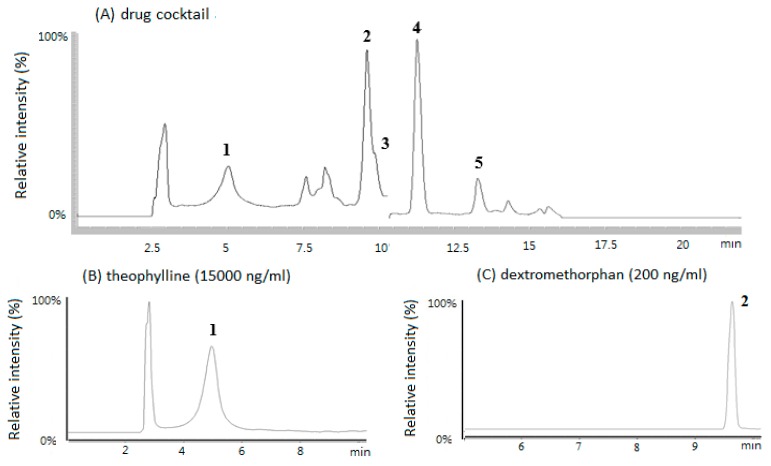

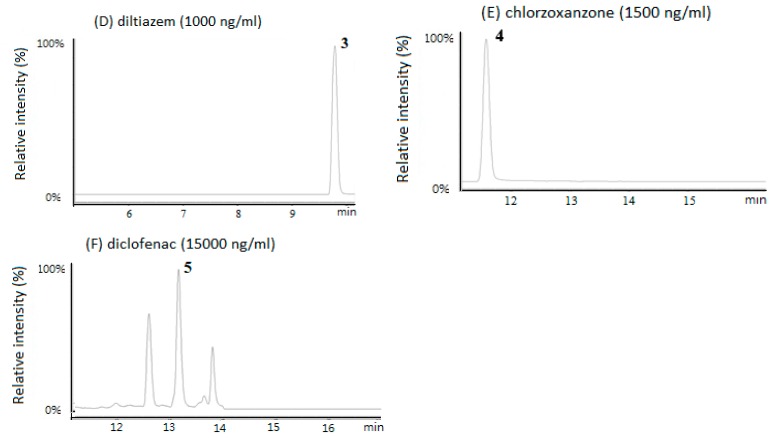
HPLC/MS (single quadrupole) selective ion monitoring chromatogram of five drugs in rat plasma (**A**), theophylline (**B**), dextromethorphan (**C**), diltiazem (**D**), chlorzoxazone (**E**), and diclofenac (**F**). Peaks: 1, theophylline; 2, dextromethorphan; 3, diltiazem; 4, chlorzoxazone; 5, diclofenac. Data acquisition was via selected ion monitoring (SIM). Details are described in Materials and Method.

Figure 1 shows the HPLC/MS chromatograms of the five CYP probe drugs in rat plasma. Each drug could be well separated and quantitated in rat plasma. The lowest limits of quantitation for theophylline, diclofenac, dextromethorphan, chlorzoxazone, and diltiazem in rat plasma were 2.0, 50, 2.67, 2.5, and 1.67 ng/mL, respectively. The calibration curves were linear over a concentration range of 100 to 15,000 ng/mL for theophylline, 50 to 15,000 ng/mL for diclofenac, 2.67 to 2000 ng/mL for dextromethorphan, 2.5 to 1500 ng/mL for chlorzoxazone, and 1.67 to 1000 ng/mL for diltiazem with correlation coefficients ≥ 0.995. The plasma concentrations of five probe drugs were analyzed by HPLC/MS that had good accuracy (greater than 90%) and precision (less than 5%) as described previously [24,29].

### 3.2. Single Oral Dose of ABO on the Pharmacokinetic Parameters of the Five Drugs after Intravenous Drug Cocktail Administration in Rats

**Figure 2:**
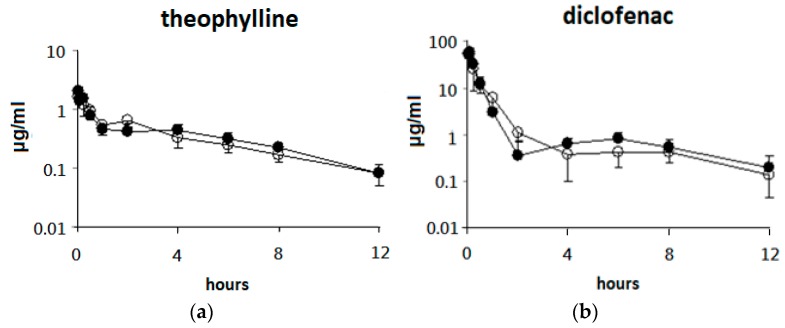

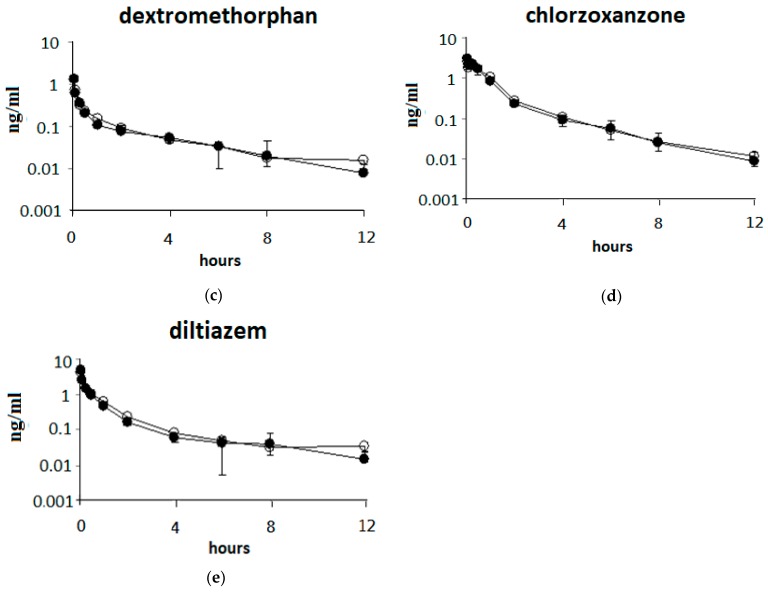
Plasma concentration–time profiles of the five drugs (IV doing) after administration of a single oral dose of adlay bran oil (ABO) in rats. Drug cocktail (CYP1A2: theophylline (**a**), CYP2C: diclofenac (**b**), CYP2D: dextromethorphan (**c**), CYP2E1: chlorzoxazone (**d**), CYP3A: diltiazem (**e**) was administered intravenously at a dose of 1–10 mg/kg body weight (BW) to rats 1 h after administration of control oil (soybean oil; 2.5 mL/kg BW) or ABO (2.5 mL/kg BW). Values at each time point are expressed as the mean ± SD of six rats in each group. ●: Control group; ○: ABO group.

The effects of pretreatment with a single oral dose of ABO on the pharmacokinetic parameters of the five drugs after intravenous drug cocktail administration in rats are presented in Figure 2 and Table 1. The results showed little or no differences in the AUC _(0–12 h)_ and t _1/2_ values between the control and ABO groups for all five drugs. These results indicated that a single oral dose of ABO did not change plasma drug concentration and CYP1A2, 2C, 2D, 2E1, and 3A activities in rat liver.

### 3.3. Single Oral Dose of ABO on the Pharmacokinetic Parameters of Five Drugs after Oral Drug Cocktail Administration in Rats

**Figure 3:**
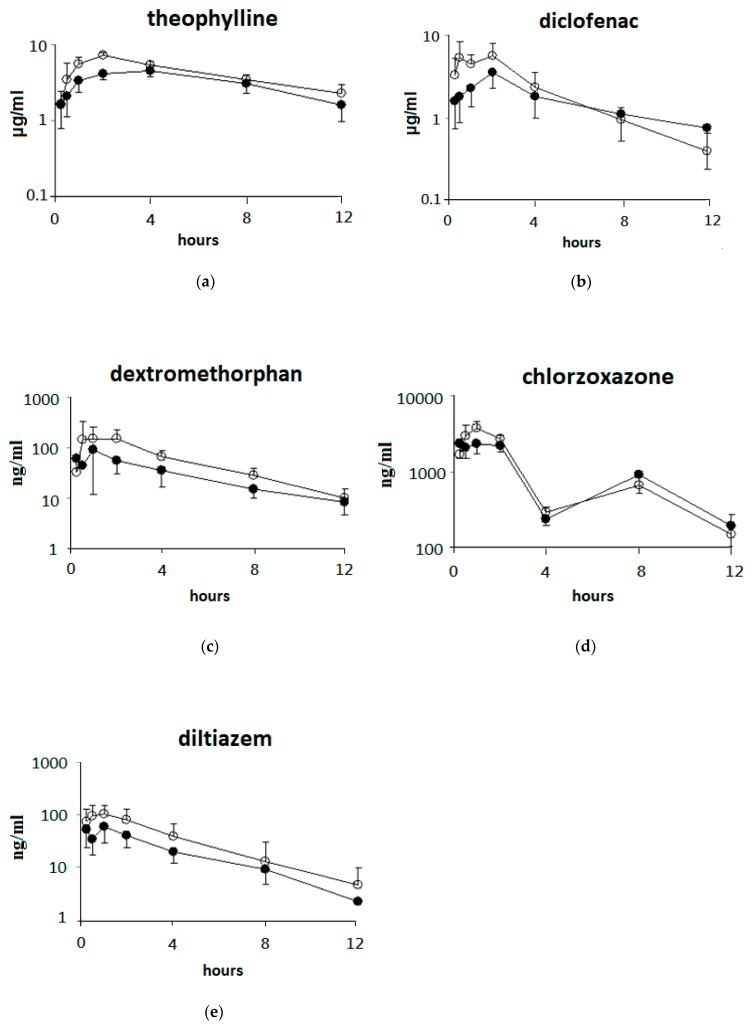
Plasma concentration–time profiles of five drugs (oral administration (PO) dosing) after administration of a single oral dose of ABO in rats. The drug cocktail (CYP1A2: theophylline (**a**), CYP2C: diclofenac (**b**), CYP2D: dextromethorphan (**c**), CYP2E1: chlorzoxazone (**d**), CYP3A: diltiazem (**e**) was administered orally at a dose of 5–40 mg/kg BW to rats 1 h after administration of 2.5 mg/kg BW of control oil or ABO. Values at each time point are expressed as the mean ± SD of six rats in each group. ●: Control group; ○: ABO group.

The effects of a single oral dose of ABO on the pharmacokinetic parameters (AUC, T_max_, and C_max_) of five drugs after oral drug cocktail administration in rats are presented in Figure 3 and Table 2. The AUC values of theophylline (+28.4%) and dextromethorphan (+48.7%) in rats were significantly increased (*p* < 0.05) by ABO. In addition, ABO caused an increase (*p* < 0.1) in the AUC value of diltiazem (+46.7%). Higher C_max_ values of theophylline (+32.4%), chlorzoxazone (+40.9%), and diltiazem (+44.3%) were observed after a single oral dose of ABO (*p* < 0.05). No significant differences in the T_max_ or t_1/2_ values of the five drugs were found between the control and ABO groups. These results indicated that pretreatment of a single oral dose of ABO may increase plasma drug levels.

### 3.4. Effects of 7 Days of ABO Feeding on the Pharmacokinetic Parameters of Five Drugs after Oral Drug Cocktail Administration in Rats 

**Figure 4:**
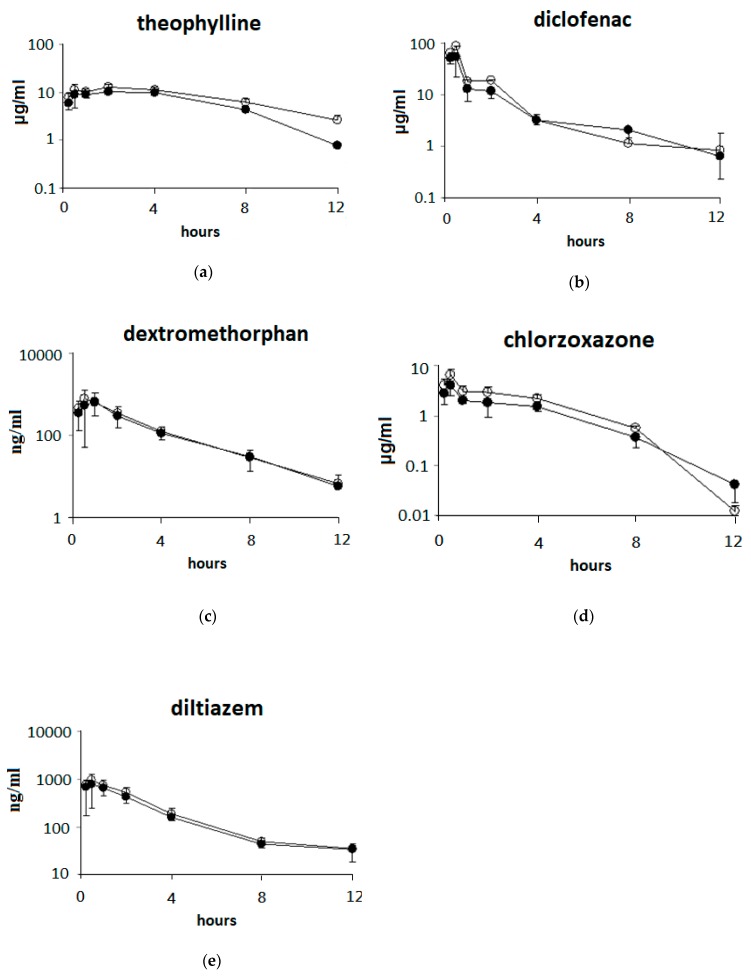
Plasma concentration–time profiles of five drugs (PO dosing) after the oral administration of soybean oil or the ABO-containing diet for 7 days in rats. The drug cocktail (CYP1A2: theophylline (**a**), CYP2C: diclofenac (**b**), CYP2D: dextromethorphan (**c**), CYP2E1: chlorzoxazone (**d**), CYP3A: diltiazem (**e**) was administered orally at a dose of 5–40 mg/kg BW to rats. Values at each time point are expressed as the mean ± SD of six rats in each group. ●: Control group; ○: ABO group.

The effects of 7 days of ABO feeding on the pharmacokinetic parameters of five drugs after oral drug cocktail administration in rats are presented in Figure 4 and Table 3. The AUC _(0–12 h)_ value of theophylline (+45.4%) in rats was significantly increased (*p* < 0.05) by ABO. In addition, higher (*p* < 0.05) AUC and C_max_ values of chlorzoxazone (+53.6%) were observed after ABO treatment (*p* < 0.05). 

### 3.5. Effects of 7 Days of ABO Feeding on Major CYP Enzyme Activities in the Liver and Intestine in Rats 

**Figure 5:**
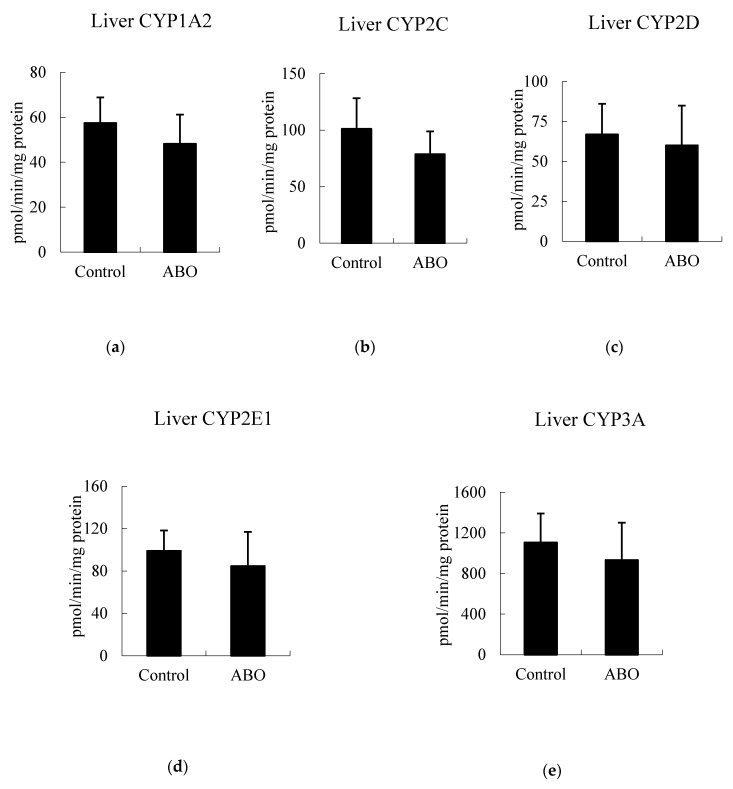

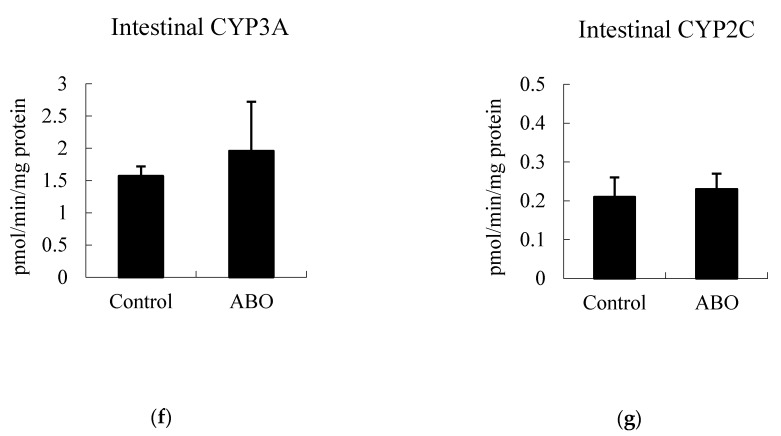
Cytochrome P450 (CYP) enzyme activities in the liver (**a–e**) and intestine (**f,g**) of rats after feeding with ABO-containing diet for 7 days.

The hepatic activities of CYP1A2, 2C, 2D, 2E1, CYP3A, and intestinal CYP3A and CYP2C activities were not changed after 7 days of ABO feeding (*p* > 0.05) (Figure 5). In this study, ABO had no significant effects on food intake or body weight gain in the control and ABO groups. In addition, ABO had no significant effects on plasma aspartate aminotransferase, alanine aminotransferase, blood urea nitrogen, or creatinine, indicating that ABO caused no hepatotoxicity or renal damage (data not shown).

## 4. Discussion

In this study, rats were given a single oral dose of ABO 1 h before intravenous or oral administration of drug cocktail to estimate the hepatic activities of five CYP isozymes and possible ABO-drug interactions. ABO did not significantly change the plasma exposure of the five CYP probe drugs when the drug cocktail was administered intravenously. These results suggest that a single oral dose of ABO may not affect CYP1A2, 2C, 2D, 2E1, and 3A activities in the liver. When a single oral dose of ABO administration or ABO feed was given for 7 consecutive days, some drug concentrations (AUC) in plasma were increased after the oral administration of the drug cocktail. These results indicated that single oral dose or short-term pretreatment with ABO may increase plasma drug levels and caused food–drug interactions. 

Many flavonoids such as nobiletin, tangeritin, rutin, and quercetin in ABO have been reported [21]. Recently, 20 phenolic compounds including 10 phenolic acids, 2 coumarins, 2 phenolic aldehydes, and 6 flavonoids in ABO were identified [31]. Some of these phytonutrients can inhibit CYP enzymes in rat and human liver microsomes [21]. However, in this study, single oral dose administration of ABO to rats had no significant effect on plasma five CYP probe drug concentrations (AUC) after intravenous administration of the drug cocktail (Table 1). This result suggested that the oral administration of ABO did not affect the five CYP enzyme activities in the liver. This observation can be explained by the fact that most of these compounds in ABO are either the result of poor absorption or extensive first-pass metabolism [32]. Indeed, in our previous study, some of these phenolic compounds in rat plasma and liver were low (<1 μM) or undetectable after 1 h of a single oral dose (2 g/kg BW) of ABO administration [21]. Thus, direct inhibition of CYP in the liver by these compounds after a single oral dose of ABO may be insignificant. 

In general, food–drug interactions involving CYP inhibitions in the gastrointestinal tract were observed when the drug was given together with foods or juices within 2 h [26]. C_max_ and T_max_ values are important parameters for the absorption phases of oral drugs [33]. In this study, pretreatment with ABO for 1 h significantly increased the plasma C_max_ of theophylline, chlorzoxazone, and diltiazem together with no difference in T_max_ values (1–2.8 h) for the five drugs when the drug cocktail was given orally (Table 2). These results indicate that oral ABO administration may enhance intestinal absorption of these drugs within the first two hours. Since a single PO dose of ABO had no effect on the five CYP enzyme activities in the liver, the increase of plasma C_max_ and AUC values of drug concentrations by ABO may have been due to its enhancement on drug absorption in the small intestine. Recently, many phytonutrients, such as quercetin, have been demonstrated to enhance the absorption of many drugs and may act as a surfactant or CYP enzyme inhibitor in the small intestine [34]. It is known that the intestinal CYP3A inhibition by some phytonutrients can be one of the important mechanisms for increasing a large proportion of oral drug absorption [10,25,34,35]. For example, pretreatment with quercetin or *Ginkgo biloba* leaf extract has been shown to increase the C_max_ and AUC of diltiazem, a typical probe of CYP3A, by inhibiting CYP3A activity in the intestinal mucosa, resulting in an increase in oral bioavailability, blood drug levels, and the efficacy of diltiazem [25,36]. Therefore, in this study, higher plasma C_max_ and AUC values of diltiazem after oral ABO administration may be partly due to high local intestinal concentrations of these phytonutrients, which could inhibit CYP3A activity [21]. In this study, however, why ABO also increased the plasma C_max_ of theophylline and chlorzoxazone is unknown because these two drugs are mainly metabolized in the liver. Therefore, it is suggested that ABO may enhance certain drug absorption in the intestinal mucosa, and, thus increasing plasma drug concentration. Further study is needed to clarify this observation. 

To further investigate whether short-term exposure (7 days) of ABO can also change plasma drug concentrations, rats were fed a 20% ABO-containing diet, which was approximately six-fold higher than the single oral dose of ABO tested. After the oral administration of the drug cocktail, increased plasma AUC of theophylline and chlorzoxazone was observed. However, the major CYP enzyme activities in the liver (CYP1A2, 2C, 2D, 2E, and 3A) [1] and small intestine (CYP3A and CYP2C) [37] were not affected by ABO treatment. Since there might have no ABO presented in the gastrointestinal tract due to overnight fasting (see protocol in animals and treatment in experiment II), therefore, the higher plasma drug concentrations after ABO feeding may not relate to its influence on CYP-mediated drug metabolism in the liver or small intestine. Thus, it is likely that ABO feeding for 7 days may reduce the expressions of the efflux pump of membrane transporters (e.g., p-glycoprotein or multidrug resistance-associated protein 2) in these tissues [35]. 

Concerning the role of the exposure time of ABO administration on CYP enzyme activity in the liver, the effects of short-term or long-term treatment may differ. In the previous study, ABO feeding (10% in the diet) for 4 weeks reduced hepatic activities of CYP1A2, 2C, 2D, 2E1, and 3A and their protein expressions [21]. It is suggested that, similar to the action of other functional foods, the modulation on CYP enzyme activity by ABO may differ with the length of intake [38,39]. Long-term ABO treatment may reduce hepatic CYP enzyme activities.

## 5. Conclusions

In conclusion, single or short-term 7 day oral administration of ABO may increase plasma drug levels without altering major CYP enzyme activities in the liver. Pretreatment of ABO may enhance drug absorption in the small intestine. Therefore, caution is needed to avoid food–drug interactions between ABO and co-administered drugs. 

## Figures and Tables

**Table 1:** Pharmacokinetic parameters of five drugs (IV dosing) after the administration of a single oral dose of ABO in rats ^1^.

Drugs	Dose (mg/kg)	AUC (0-t) ^2^ (μg/mL×h)	*T _1/2_* (h)	Cl ^3^ (mL/min/kg)
**Theophylline (CYP1A2)**				
Control group	1	4.6 ± 0.8	3.2 ± 1.1	12.7 ± 2.3
ABO group		4.5 ± 0.7	3.7 ± 0.6	12.5 ± 1.7
**Diclofenac (CYP2C)**				
Control group	10	30.1 ± 3.2	2.9 ± 2.1	19.2 ± 2.3
ABO group		28.7 ± 6.7	2.6 ± 2.1	20.5 ± 5.3
**Dextromethorphan (CYP2D)**				
Control group	5	0.76 ± 0.17	2.4 ± 0.7	388.4 ± 65.8
ABO group		0.89 ± 0.08	4.2 ± 3.7	318.4 ± 27.9
**Chlorzoxazone (CYP2E1)**				
Control group	1	3.0 ± 0.6	1.7 ± 0.6	20.1 ± 4.3
ABO group		3.0 ± 0.7	2.4 ± 1.0	18.1 ± 2.9
**Diltiazem (CYP3A)**				
Control group	5	5.1 ± 0.7	2.2 ± 1.4	128.9 ± 16.6
ABO group		4.3 ± 1.2	1.9 ± 0.3	115.3 ± 13.9

^1^ Drug cocktail was administered intravenously at a dose of 1–10 mg/kg BW to rats 1 h after the administration of control oil or ABO. Pharmacokinetic parameters were expressed as the mean ± SD of six rats in each group. ^2^ AUC: area under the plasma drug concentration curve. *t* = 12 h. ^3^ Cl: Clearance, the volume of plasma from which a drug is removed per unit time.

**Table 2:** Pharmacokinetic parameters of five drugs (PO dosing) after the administration of a single oral dose of ABO in rats ^1^.

Drugs	Dose (mg/kg)	AUC (0-t) ^2^ (μg/mL×h)	T_max_^3^ (h)	C_max_^4^ (μg/mL)	*t_1/2_*^5^ (h)	F% ^6^
**Theophylline (CYP1A2)**						
Control group	10	52.9 ± 15.9	2.8 ± 1.3	4.8 ± 0.7	5.6 ± 1.5	86.0 ± 15.9
ABO group		73.9 ± 17.2 *	2.0 ± 0.0	7.1 ± 0.9 *	6.5 ± 1.9	124.8 ± 14.7 *
**Diclofenac (CYP2C)**						
Control group	20	22.2 ± 7.9	1.5 ± 0.7	4.7 ± 2.2	4.1 ± 1.6	29.2 ± 8.1
ABO group		27.1 ± 8.5	1.3 ± 0.8	6.7 ± 3.0	2.5 ± 0.8	40.5 ± 12.1
**Dextromethorphan (CYP2D)**						
Control group	25	0.40 ± 0.15	1.2 ± 0.6	0.13 ± 0.1	3.1 ± 0.5	10.0 ± 2.8
ABO group		0.78 ± 0.23 *	1.5 ± 0.6	0.22 ± 0.2	3.1 ± 0.8	18.6 ± 6.8 *
**Chlorzoxazone (CYP2E1)**						
Control group	5	12.6 ± 2.7	1.3 ± 0.8	2.8 ± 0.6	4.1 ± 1.9	76.2 ± 11.5
ABO group		13.0 ± 2.3	1.3 ± 0.6	3.8 ± 0.9 *	2.7 ± 1.1	78.1 ± 11.3
**Diltiazem (CYP3A)**						
Control group	40	0.24 ± 0.04	1.3 ± 0.8	0.073 ± 0.028	2.0 ± 0.7	1.3 ± 0.1
ABO group		0.45 ± 0.22 ^#^	1.0 ± 0.8	0.131 ± 0.045 *	2.0 ± 0.8	2.2 ± 1.0

^1^ Drug cocktail was administered intravenously at a dose of 5–40 mg/kg BW to rats 1 h after the administration of control oil (soybean oil; 2.5 mL/kg BW) or ABO (2.5 mL/kg BW). Pharmacokinetic parameters were expressed as the mean ± SD of six rats in each group. ^2^ AUC: area under the plasma drug concentration curve. t = 12 h. ^3^ T_max_: the time at which maximum concentration is observed. ^4^ C_max_: values of maximal observed concentration. ^5^ t_1/2_: half-life, the time required for the amount of drug in the body to decrease by half. ^6^ F% = (AUC_PO(0−12h)_/PO dose) ×100/(AUC_IV(0−12h)_/IV dose). Oral bioavailability of the theophylline calculated by a computer fitting method was approximately or greater than 100%, suggesting that the theophylline was completely absorbed [30]. * Significantly different from the control group, *p* < 0.05. ^#^ Significantly different from the control group, *p* < 0.1.

**Table 3:** Pharmacokinetic parameters of five drugs (PO dosing) after feeding with the ABO-containing diet for 7 days in rats ^1^.

Drugs	Dose (mg/kg)	AUC (0-t) ^2^ (μg/mL×h)	C_max_^3^ (μg/mL)	T_max_^4^ (h)	*t_1/2_*^5^ (h)
**Theophylline (CYP1A2)**					
Control group	10	78.6 ± 8.3	11.4 ± 2.9	2.1 ± 1.2	2.1 ± 1.0
ABO group		114.3 ± 15.9 *	13.6 ± 2.6	2.1 ± 1.2	4.0 ± 0.9 *
**Diclofenac (CYP2C)**					
Control group	20	84.8 ± 12.4	65.9 ± 35.3	0.4 ± 0.1	2.5 ± 1.0
ABO group		106.1 ± 28.3	87.0 ± 31.8	0.5 ± 0.0	1.5 ± 0.3
**Dextromethorphan (CYP2D)**					
Control group	25	1.7 ± 0.8	0.76 ± 0.44	0.8 ± 0.3	1.8 ± 0.5
ABO group		1.9 ± 1.0	0.84 ± 0.49	0.8 ± 0.6	1.8 ± 0.3
**Chlorzoxazone (CYP2E1)**					
Control group	5	12.5 ± 0.5	4.0 ± 1.6	0.8 ± 0.6	1.5 ± 0.3
ABO group		19.2 ± 3.7 *	6.7 ± 2.4 *	0.5 ± 0.0	1.2 ± 0.2
**Diltiazem (CYP3A)**					
Control group	40	1.0 ± 0.6	0.81 ± 0.59	0.5 ± 0.3	1.2 ± 0.1
ABO group		1.4 ± 0.9	0.94 ± 0.74	0.8 ± 0.7	1.3 ± 0.3

^1^ Drug cocktail was administered orally at a dose of 5–40 mg/kg BW to rats after administration of control or 20% ABO diet. Plasma drug concentrations were expressed as the mean ± SD of six rats in each group. ^2^ AUC: area under the plasma drug concentration curve. t = 12 h. ^3^ C_max_: values of maximal observed concentration. ^4^ T_max_: the time at which maximum concentration is observed. ^5^ t_1/2_: elimination half-life; the time required for the amount of drug in the body to decrease by half. * Significantly different from the control group, *p* < 0.05.
